# Comparison of the Efficacy of Atorvastatin with Rosuvastatin in Preventing Cardiovascular Events Among Patients With Cardiovascular Disease: A Meta-Analysis

**DOI:** 10.7759/cureus.50421

**Published:** 2023-12-12

**Authors:** Calvin R Wei, Fahad Lakhdhir, Anurag Rawat, Abraham K Isaak, Areeba Riaz, Mohammad Al Omari, Revanth Reddy Bandaru, Adil Amin

**Affiliations:** 1 Research and Development, Shing Huei Group, Taipei, TWN; 2 Adult Cardiology, National Institute of Cardiovascular Diseases, Karachi, PAK; 3 Interventional Cardiology, Himalayan Institute of Medical Sciences, Dehradun , IND; 4 Telemetry, Sharp Memorial Hospital, San Diego, USA; 5 Internal Medicine, Orotta School of Medicine and Dentistry, Asmara, ERI; 6 Medicine, Quiad-e-Azam Medical College, Bahawalpur, PAK; 7 Medicine, Yarmouk University, Irbid, JOR; 8 Internal Medicine, East Carolina University, Greenville, USA; 9 Cardiology, Pakistan Navy Ship (PNS) Shifa, Karachi, PAK

**Keywords:** meta-analysis, cardiovascular event, rosuvastatin, atorvastatin, efficacy

## Abstract

The aim of this study was to assess and compare the efficacy of atorvastatin with rosuvastatin in preventing cardiovascular events among patients already diagnosed with cardiovascular disease (CVD). We performed this systematic review and meta-analysis as per the Preferred Reporting Items for Systematic Reviews and Meta-Analysis (PRISMA) guidelines. Two investigators independently searched online databases, including PubMed, the Cochrane Library, and the Excerpta Medica database (Embase), from the inception of databases until November 2023. The primary outcome assessed in the meta-analysis included cardiovascular mortality and a composite of cardiovascular events. Other outcomes included myocardial infarction and stroke.

A total of four studies were selected for our meta-analysis. A total of 7,378 patients were enrolled, including 3,721 in the atorvastatin group and 3,657 in the rosuvastatin group. Pooled analysis showed that the incidence of composite cardiovascular events was not significantly different in patients receiving atorvastatin and patients receiving rosuvastatin (risk ratio (RR): 0.93, 95% confidence interval (CI): 0.79 to 1.09, p-value: 0.38, I-square: 0%). Pooled analysis showed that the risk of cardiovascular mortality was not significantly different between the two study groups (RR: 0.96, 95% CI: 0.51 to 1.81, p-value: 0.93, I-square: 0%). In conclusion, our meta-analysis, based on four selected studies, found no significant disparities in composite cardiovascular events, cardiovascular mortality, myocardial infarction, or stroke between patients administered atorvastatin and those receiving rosuvastatin. This outcome underscores the comparable efficacy of these statins in mitigating cardiovascular risks, highlighting their clinical equipoise in the realm of secondary prevention.

## Introduction and background

As per the World Health Organization (WHO), cardiovascular diseases (CVDs) stand as a prominent global cause of mortality. Substantial efforts have been exerted to diminish the associated mortality and morbidity [1]. The primary focus of managing CVD lies in drug therapy, and various treatment options are available. Notably, statin therapy has demonstrated a mortality benefit for individuals with confirmed coronary artery disease (CAD) [2-3]. In line with the 2018 and 2013 guidelines on dyslipidemia by the American Heart Association/American College of Cardiology (AHA/ACC), individuals aged 75 or below with atherosclerotic cardiovascular disease (ASCVD) should undergo high-intensity statin therapy as a secondary prevention, aiming for a 50% reduction in low-density lipoprotein cholesterol (LDL-C) [3].

Atorvastatin and rosuvastatin, both belonging to the 3-hydroxy-3-methylglutaryl coenzyme A (HMG-CoA) reductase inhibitor class, are frequently prescribed statins. These medications play a crucial role in controlling elevated cholesterol levels, thereby mitigating the risk of cardiovascular diseases. Atorvastatin is acknowledged for its effectiveness in lowering LDL-C, while rosuvastatin is commended for its potency and its ability to raise high-density lipoprotein (HDL) cholesterol [4]. Physicians typically make a choice between these two based on individual patient requirements and response to treatment. Although prior studies have assessed clinical outcomes with varying statin intensities for managing dyslipidemia in individuals with coronary artery disease, there has been insufficient examination of the effects of different statin types [5-6]. Apart from the demonstrated efficacy of statins in reducing LDL-C levels and averting future adverse cardiovascular events, it is crucial to consider safety concerns such as statin-related adverse effects and intolerance in practical, real-world settings [7-8].

The presumption of comparable clinical benefits is grounded in their capacity to lower lipid levels and the demonstrated clinical advantages of rosuvastatin in individuals undergoing primary prevention. However, given that those in secondary prevention exhibit distinct clinical characteristics, such as a higher prevalence of diabetes [9], vascular revascularization [9], and varied concurrent medications compared to those in primary prevention, it is important to ascertain whether both statins yield similar benefits in real-world secondary prevention scenarios. To investigate potential variations in the clinical response to atorvastatin and rosuvastatin in individuals with cardiovascular disease, we conducted a meta-analysis that encompassed both randomized controlled trials (RCTs) and observational studies. This study aimed to assess and compare the efficacy of atorvastatin and rosuvastatin in preventing cardiovascular events among patients already diagnosed with CVD.

## Review

Methodology

We performed this systematic review and meta-analysis as per the Preferred Reporting Items for Systematic Reviews and Meta-Analysis (PRISMA) guidelines. The meta-analysis is registered with the International Prospective Register of Systematic Reviews (PROSPERO) (registration number: CRD42023468458).

Literature Search Strategy

Two investigators independently searched online databases, including PubMed, the Cochrane Library, and the Excerpta Medica database (Embase), from the inception of databases to November 2023. The keywords used to search for relevant articles included "rosuvastatin," "atorvastatin," “cardiovascular outcomes,” and “cardiovascular disease,” along with their synonyms and incorporating medical subject heading terms (MeSH) and Boolean operators. Other sources of data were also searched, including relevant reviews from major medical journals, unpublished and unprinted articles, conference papers, and bibliographies of editorials. Additionally, a reference list of all articles was manually screened to find additional studies relevant to the study topic.

Study Design and Selection Criteria

The method for determining eligibility and decision-making for including or excluding studies was hierarchical, based on an initial review of titles and abstracts followed by full-text screening. We followed the Joanna Briggs Institute’s (JBI) protocol for critical appraisal and study selection, which provides more rigorous and specific criteria for the process of study selection. The studies which had the following features were included: 1) performed on individuals aged 18 years or older and with CVD; 2) were RCTs and observational studies (retrospective and prospective cohort); and 3) reported one of the outcomes assessed in this meta-analysis. The pre-defined exclusion criteria included the following: 1) studies with a lack of a control group; 2) original studies, including case series and case reports; 3) studies that involved animals; and 4) reviews, meta-analyses, and editorials.

Data Extraction and Assessment of Quality

The systematic search yielded articles that were subsequently imported into EndNote X9 Reference Manager (Clarivate Analytics, Philadelphia, PA). We removed duplicate entries across various online databases. Two independent researchers conducted a comprehensive assessment of the remaining articles. Only studies meeting predetermined inclusion criteria were retained. Initially, titles and abstracts of all studies underwent screening, followed by a thorough examination of the full text to assess relevance. Any disparities were resolved through discussion with the principal investigator. Data collection encompassed study characteristics such as name of author, publication year, region, study design, and follow-up duration. Population characteristics, including sample size, mean age, number of males, and baseline comorbidities like diabetes and hypertension (HTN), were also extracted. The primary outcome assessed in the meta-analysis included cardiovascular mortality and a composite of cardiovascular events. Other outcomes included myocardial infarction and stroke.

Statistical Analysis

We performed statistical analysis using Review Manager (RevMan) (computer program), version 5.4.1, The Cochrane Collaboration, 2020). The results were presented as a risk ratio (RR) with a 95% confidence interval (CI) and pooled using an inverse variance-weighted random effects model. A p-value ≤0.05 was considered significant in all cases. I-square was used to categorize heterogeneity as low (<25%), moderate (25-20%), and high (>50%).

Results

**Figure 1:**
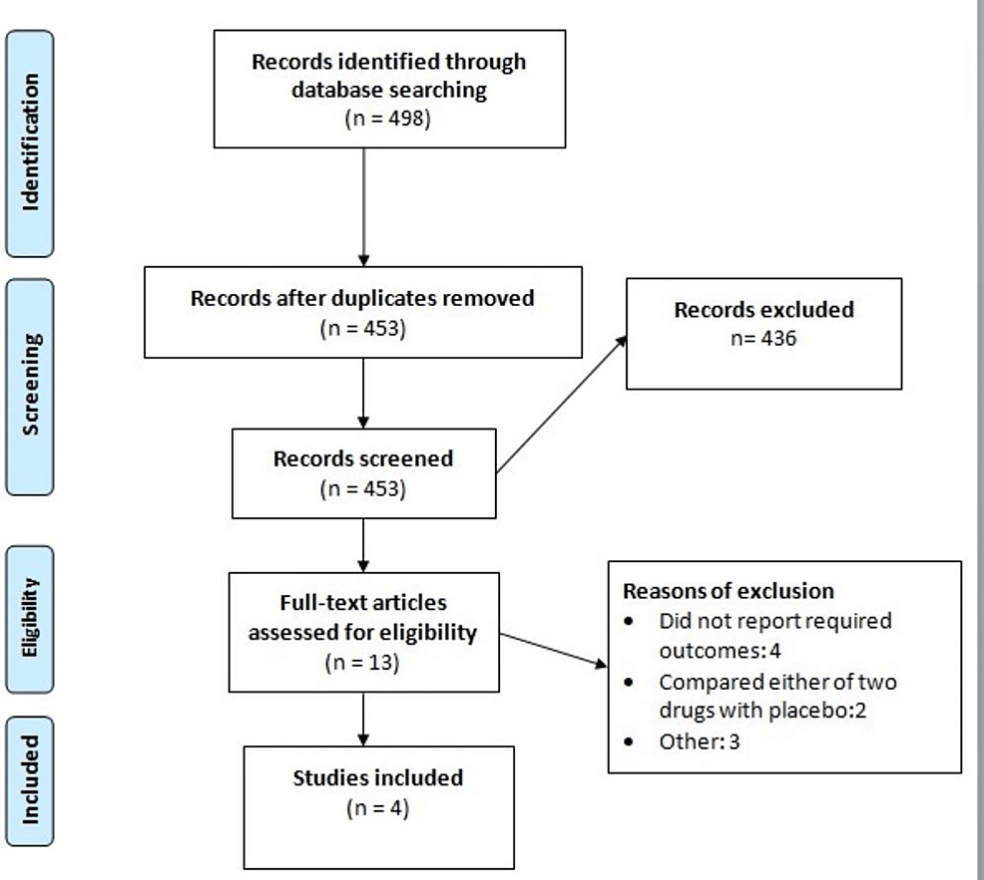
A PRISMA flowchart outlining the study selection process PRISMA: Preferred Reporting Items for Systematic Reviews and Meta-Analyses

Our initial search yielded 498 records, out of which 453 studies remained after removing the duplicates. Screening based on abstract and title resulted in the exclusion of 436 studies. After screening the full text for eligibility, 13 studies were excluded. As a result, four studies were selected for our meta-analysis. The PRISMA flow diagram in Figure 1 illustrates the complete literature search procedure.

Detailed study and population characteristics of the included studies are given in Table 1.

**Table 1:** Characteristics of the included studies RC: retrospective cohort; RCT: randomized controlled trial

Author name	Year	Study design	Groups	Sample size	Follow-up	Age	Males (n)	Hypertension (n)	Diabetes (n)
Calahorra et al. [10]	2019	RC	Atorvastatin	210	36 Months	60.9	190	118	75
			Rosuvastatin	135		60.6	130	92	52
Lee et al. [11]	2023	RCT	Atorvastatin	2196	36 Months	65	1570	1439	743
			Rosuvastatin	2204		65	1602	1498	725
Nicholls et al. [12]	2011	RCT	Atorvastatin	689	26 Months	57.9	386	367	87
			Rosuvastatin	691		57.4	379	364	72
Rahhal et al. [13]	2022	RC	Atorvastatin	626	12 Months	50	606	254	296
			Rosuvastatin	627		52	594	242	286

A total of 7,378 patients were enrolled, including 3,721 in the atorvastatin group and 3,657 in the rosuvastatin group. The follow-up of the included studies ranged from 12 to 36 months.

Results of the Meta-Analysis

**Figure 2:**
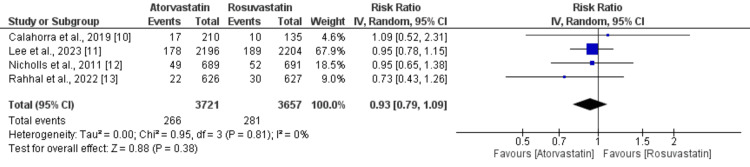
A comparison of the composite cardiovascular events between the two groups

Primary outcomes: Composite cardiovascular events were reported by four studies, as shown in Figure 2.

**Figure 3:**
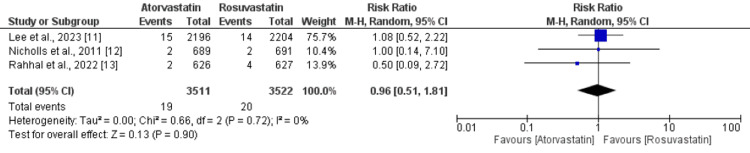
A comparison of composite cardiac-related mortality between the two groups

Pooled analysis showed that the incidence of composite cardiovascular events was not significantly different in patients receiving atorvastatin and patients receiving rosuvastatin (RR: 0.93, 95% CI: 0.79 to 1.09, p-value: 0.38, I-square: 0%). A total of three studies provided data on cardiovascular mortality among patients with cardiovascular diseases, as shown in Figure 3.

Pooled analysis showed that the risk of cardiovascular mortality was not significantly different between the two study groups (RR: 0.96, 95% CI: 0.51 to 1.81, p-value: 0.93, I-square: 0%).

**Figure 4:**
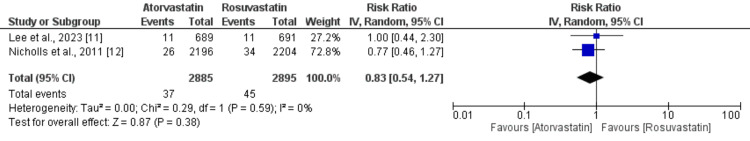
A comparison of the risk of myocardial infarction between the two groups

Secondary outcomes: A pooled analysis of two studies reported insignificant differences in myocardial infarction in patients receiving atorvastatin and rosuvastatin (RR: 0.83, 95% CI: 0.54 to 1.27, p-value: 0.38, I-square: 0%), as shown in Figure 4.

**Figure 5:**
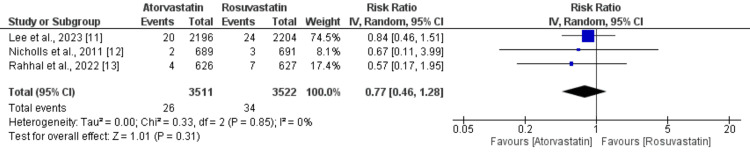
A comparison of the risk of stroke between the two groups

A total of three studies compared the effects of atorvastatin and rosuvastatin on the risk of stroke, and the results are shown in Figure 5.

The pooled analysis revealed that the incidence of stroke was not significantly different between the two groups (RR: 0.77, 95% CI: 0.46 to 1.28, p-value: 0.31, I-square: 0%).

Discussion

Our meta-analysis aimed to compare the cardiovascular outcomes between patients who received atorvastatin and patients who received rosuvastatin. The pooled analysis of four studies revealed that the risk of composite cardiovascular events, cardiovascular mortality, myocardial infarction, and stroke was not significantly different between patients who received atorvastatin and patients who received rosuvastatin.

The observed similarity in efficacy between atorvastatin and rosuvastatin in preventing CVD can be attributed to their shared mechanism of action and potent cholesterol-lowering abilities [14]. Both drugs belong to the statin class and work by inhibiting HMG-CoA reductase, a key enzyme in the cholesterol synthesis pathway. This inhibition leads to a reduction in circulating LDL-C levels, which is a major contributor to atherosclerosis and cardiovascular events [15].

In clinical settings, the selection of an appropriate statin type and intensity is crucial. Notably, rosuvastatin and atorvastatin stand out as the only options capable of providing both high- and moderate-intensity statin treatments, typically necessary for individuals with cardiovascular disease aiming to significantly reduce their LDL-C levels [16-17]. Previous studies have demonstrated the clinical benefits of employing either of these potent statins in individuals with CAD [5]. The efficacy of rosuvastatin in primary cardiovascular event prevention is well established. For example, the JUPITER trial (an acronym for the Justification for the Use of Statins in Primary Prevention: An Intervention Trial Evaluating Rosuvastatin Trial), involving over 17,000 healthy volunteers with LDL-C levels below 130 mg/dL, investigated the impact of rosuvastatin compared to a placebo for primary prevention [18]. The trial, terminated after 1.9 years of follow-up, revealed a significant reduction in major cardiovascular events, such as myocardial infarction, stroke, arterial revascularization, hospitalization for unstable angina, and cardiovascular-related death with rosuvastatin use compared to the placebo (hazard ratio (HR) = 0.56; 95% CI, 0.46-0.69; p <0.001) [18]. However, the evidence supporting the benefits of rosuvastatin in cardiovascular secondary prevention is limited, with most literature focusing on high-intensity atorvastatin. Currently, there is a scarcity of studies comparing rosuvastatin and atorvastatin for secondary prevention based on cardiovascular clinical outcomes. To address this gap, two virtual trials using the Archimedes model, an individual-based simulation of human pathophysiology and treatment intervention, were conducted to assess and compare the clinical outcomes of rosuvastatin versus atorvastatin in cardiovascular secondary prevention [19-20].

Our meta-analysis consolidates evidence from four studies, revealing no significant difference in composite cardiovascular events, cardiovascular mortality, myocardial infarction, or stroke between patients receiving atorvastatin and those receiving rosuvastatin. This finding suggests comparable efficacy in mitigating cardiovascular risks, emphasizing the clinical equipoise between the two statins in the context of secondary prevention. The absence of a statistically significant difference underscores the potential interchangeability of these agents in tailoring treatment strategies for individuals with cardiovascular disease. Clinicians may consider patient-specific factors, tolerability, and cost implications when deciding between atorvastatin and rosuvastatin for secondary prevention.

The present meta-analysis faces limitations, comprising only four studies, with two being RCTs, necessitating cautious interpretation of findings. The absence of individual-level data precluded subgroup analyses, hindering the exploration of differential effects in specific groups, such as diabetes versus non-diabetes. Moreover, safety events were only analyzed in one study. Therefore, we were not able to assess these outcomes in the present meta-analysis. Future trials comparing atorvastatin and rosuvastatin for secondary prevention are imperative to augment the evidence base. A more extensive study pool would enhance generalizability, while individual-level data would facilitate nuanced subgroup analyses. Addressing these limitations is crucial for advancing our understanding of the comparative efficacy of atorvastatin and rosuvastatin in preventing cardiovascular events in secondary prevention scenarios.

## Conclusions

In conclusion, our meta-analysis, based on four selected studies, found no significant disparities in composite cardiovascular events, cardiovascular mortality, myocardial infarction, or stroke between patients administered atorvastatin and those receiving rosuvastatin. This outcome underscores the comparable efficacy of these statins in mitigating cardiovascular risks, highlighting their clinical equipoise in the realm of secondary prevention. Given these findings, clinicians can potentially consider both atorvastatin and rosuvastatin as interchangeable options when tailoring treatment strategies for individuals with cardiovascular disease. The decision-making process should account for patient-specific factors, tolerability, and cost considerations.
